# Measurement of hepatic glucose (^18^F-fluorodeoxyglucose) uptake with positron emission tomography-magnetic resonance imaging in fumonisin B intoxicated rabbit bucks

**DOI:** 10.1038/s41598-024-68210-3

**Published:** 2024-08-06

**Authors:** András Szabó, Miklós Emri, Zoltán Tóth, Dániel Fajtai, Tamás Donkó, Örs Petneházy, Dénes Kőrösi, Imre Repa, Alíz Takács, Tímea Kisiván, Zsolt Gerencsér, Omeralfaroug Ali, Janka Turbók, Brigitta Bóta, Patrik Gömbös, Róbert Romvári, Melinda Kovács

**Affiliations:** 1 Agribiotechnology and Precision Breeding for Food Security National Laboratory, Department of Physiology and Animal Health, Institute of Physiology and Nutrition, Hungarian University of Agriculture and Life Sciences, Kaposvár, Hungary; 2 HUN-REN-MATE Mycotoxins in the Food Chain Research Group, Kaposvár, Hungary; 3 Division of Nuclear Medicine and Translational Imaging, Department of Medical Imaging, Faculty of Medicine, University of Debrecen, Debrecen, Hungary; 4 Medicopus Healthcare Provider and Public Nonprofit Ltd, Somogy County Moritz Kaposi Teaching Hospital, Kaposvár, Hungary; 5 Department of Animal Breeding, Institute of Animal Sciences, Hungarian University of Agricultural and Life Sciences, Kaposvár, Hungary; 6 National Food Chain Safety Office, Animal Health Directorate, Animal Health Diagnostic Laboratory, Kaposvár, Hungary

**Keywords:** Rabbit, Liver, Fumonisin B, Positron emission tomography, ^18^F-fluorodeoxyglucose, Glucose uptake, Molecular medicine, Toxicology

## Abstract

Rabbit bucks (bodyweight 5 kg) underwent dietary intoxication with fumonisin B series mycotoxins (FB_1_ + FB_2_ + FB_3_, 15 mg/kg diet) for 14 days to test the applicability of positron emission tomography-magnetic resonance (PET MR) hybrid imaging in characterizing experimentally induced mild hepatotoxicosis. ^18^F-fluorodeoxyglucose (^18^F-FDG) radiotracer-aided imaging was performed before and after FBs administration on identical animals, and at both time points, blood was sampled for haematology and clinical chemistry. Kinetic PET image analysis revealed time-activity curves with uptake maxima below 1 min in the liver, renal cortex, portal vein, lung and coarctatio aortae. In the frame of static PET image analysis, based on the standardized uptake value (SUV), the so-called metabolic liver volume (MLV, liver volume defined by over 0.9 × average liver SUV) and the total liver glycolysis (TLG, MLV multiplied by the SUVmean) were calculated. Mycotoxicosis increased total liver glycolysis (p < 0.04) after 14 days and liver tissue TLG inhomogeneity was minimal. Pearson correlation between TLG and alkaline phosphatase (ALP) was positive (0.515), while negative with LDH and AST (− 0.721 and − 0.491, respectively). Results indicate a slight hepatic mycotoxin effect and significantly increased glucose uptake intensity, which has been sensitively detected with molecular imaging (^18^F-FDG PET MRI) in the rabbit model.

## Introduction

*Fumonisins* are a series of structurally related fungal toxins (indeed secondary metabolites) produced by *Fusarium verticillioides* and *proliferatum* filamentous fungi (*Liseola* section), mostly infecting maize and other cereal commodities^1^. Among the fumonisin series A, B, C and P, the B analogues are toxicologically the most hazardous, with fumonisin B_1_ (FB_1_) being the most well-known and the most toxic. There are more than 28 FB_1_ isomers characterized^2^. Fumonisin occurrence is primarily frequent in maize commodities, but may also occur on other crops and food items^3,4^; the prevalence was 68% in 2022 in the tested corn samples^5^. The EU Commission Regulation^6^ on the maximum allowed levels for FB_1+2_ in unprocessed maize kernels is 4 mg/kg (food and feed also), while for rabbit feed this is 5 mg/kg^7^.

*The harmful effects* of FB_1_ are species-specific in vertebrates^8,9^. FB_1_-induced hepatotoxic effects in rabbits have been reported at 7.5–10 mg/kg feed doses for 196 days (Ewuloa, 2009) and also at 10 mg/kg for 4 weeks^10^. The mode of action is mostly well understood^9^, providing organ specificity even within rodents^9^, with the liver and/or kidney being the target organs. FB_1_ is a ceramide conformational analogue and a competitive inhibitor of CoA-dependent ceramide synthase^11^. Harmful effects of FB_1_ in the liver and kidney of most domestic animal species are exerted via alterations of the sphingolipid metabolism, ultimately causing apoptosis and oncotic necrosis, and as well carcinogenesis in rodents^9^.

*Fumonisins' effects on the liver* are quite widespread^9^. The in vivo hepatic effects are based on two toxic actions: the drastic disruption of lipid metabolism and the induction of further molecular changes leading to diseases/malfunctions. Lipids undergoing quantitative or qualitative changes are per se primarily the sphingolipids^12^, as FB_1_ not only blocks the biosynthesis of complex sphingolipids, but induces sphinganine accumulation. According to Riley et al.^9^, sphinganine is partly metabolized to sphinganine-1-phosphate and degraded to hexadecanal and ethanolamine phosphate; the latter is incorporated into phosphatidylethanolamine (PE). Quantitative alterations of the hepatic PE (and P-choline) lipid fraction were shown in vitro in primary rat hepatocytes^13^, while in vivo*,* our team provided evidence for the perturbation of the fatty acid metabolism of PE and PC fractions in rats^14^. The facts above support that FBs are inducers of a complex change in multiple lipid fractions’ composition^14^, some of them being involved in the regulation of energy metabolism^15^.

With regards to *glucose metabolism*, in the development of insulin resistance (either in the liver or muscle), bioactive lipids play determinant roles, with ceramides being one the most potent inhibitors of insulin signal transduction^16^. Meanwhile, hepatic glucose intolerance is primarily associated with increased diacyl-glycerol levels. The inter-relationship between hepatic tissue ceramide level and glucose intolerance has also been highlighted in the skeletal muscle^17^. According to Babenko and Kharchenko^18^, stress (and aging) associated modification (decrease) of hepatic ceramide levels/sphingolipid metabolism partly improved insulin regulation of glucose metabolism in vitro in rat hepatocytes. Ceramide synthesis inhibitors (e.g. fumonisin B_1_) improved insulin-mediated glucose metabolism^18^, thus enhancing cellular insulin-induced glucose uptake. As a minor addition, it shall be noted that in vivo fumonisin feeding studies often report the alterations in plasma glucose levels, more specifically a decrease (supporting insulin-mediated glucose uptake of the liver and muscles), as observed in chicks^19^ and heifers^20^, co-exposure with aflatoxin). While in vitro, in porcine intestinal cells (IPEC-J2), FB_1_ reduced the expression of glucose transporters, Na^+^/glucose co-transporter 1 (SGLT1) and glucose transporter 2 (GLUT2)^21^, in vivo Na-dependent glucose absorption is up-regulated in pigs after acute or longterm exposure to FB_1_^22^. Our hypothesis was set up on the above basis, namely fumonisin B_1_ is a characteristic ceramide synthesis inhibitor^23^, and ceramide efficiently lowers insulin sensitivity. Thus, the reduction of hepatic ceramide level shall—theoretically—up-regulate hepatic glucose uptake.

^*18*^*F-fluorodeoxyglucose positron emission tomography* (^18^F-FDG PET) is a reliable, functional imaging tool that provides valuable information for detecting, staging, and evaluating the therapeutic responses^24^, applicable also on small pet animals such as rabbits^25^. As a glucose analogue, the uptake of ^18^F-FDG is a biological process of glucose consumption at cellular levels through intra-cellular transportation and phosphorylation. Since the liver is one of the organs in the body with relatively intensive glucose metabolism, ^18^F-FDG is taken up by liver cells. Various factors, including toxicity, can influence this uptake, such as certain medications or diseases (hepatitis, fatty liver disease, cirrhosis, infections, and tumours). Our study focused exclusively on toxicity in a controlled cohort of rabbits, utilizing PET MRI imaging with ^18^F-FDG. We gathered accurate anatomical information and tracked the uptake stage and changes of ^18^F-FDG by using a bimodal PET MRI scanner, which provided simultaneously acquired MRI and kinetic PET images^26^.

To test and possibly quantify the fumonisin B series’ effect on the hepatic glucose uptake (and its alterations), we used a rodent model of known hepatic reaction for this intoxication^23^. We invented a coupled/hybrid imaging technique (PET MR imaging) for analyzing hepatic glucose uptake in not severely fumonisin-intoxicated adult rabbit bucks.

## Material and methods

### Animals

Rabbit bucks of ca. 5 kg bodyweight (n = 4) of the Pannon White breed were enrolled in the study. Animals were kept individually in cages, and were fasted for exactly 6 h before the scanning protocol initiation to reach a nearly euglycaemic status (mean ± SD of the 1st and 2nd timepoints: 7.48 ± 0.88 and 7.21 ± 0.66 mmol/L, p = 0.57)^27^, leading to possibly uniform glucose baseline values on which tracer glucose was superposed. All animals were individually marked and scanned twice, before and after mycotoxin exposure, i.e. serving as their own individual controls. The bodyweight of animals was measured weekly, individually during the study (Fig. 2).

### Feeding and intoxication

A total exposure period of 14 days was performed. A commercial rabbit feed (composition: Table 1) was mixed with a fungal culture containing fumonisin B series. The FBs (FB_1_ + FB_2_ + FB_3_) feed concentration was 15 mg/kg. The mycotoxin concentration of the control and the experimental feed was determined with LC–MS^28^. The limit of detection (LOD) for FB_1_ was 3 µg/kg. The diet fed to the control group did not contain detectable amounts of FBs (the full absence of deoxinivalenol, zearalenone and T-2 toxin was as well controlled and confirmed). Feed consumption was measured individually (Fig. 2).

**Table 1:** Chemical composition of the diet.

Chemical composition	
Dry material (%)	89
Crude protein (%)	14.5
Ether extract (%)	2.4
Crude fibre (%)	17.1
Ash (%)	7.5
Lysine (%)	0.9
Methionine (%)	0.41
Calcium (%)	0.88
Phosphorus (%)	0.52
Sodium (%)	0.19
Vitamin A (IU/kg)	14,000
Vitamin D3 (IU/kg)	1300
Vitamin E (mg/kg)	107
Digestible energy (MJ/kg)	9.7

### Haemogram and clinical chemistry

Whole blood smears were May-Grünwald-Giemsa stained, covered using Entellan mounting medium (Merck 1.07960) and blood cells were evaluated with light microscopy (visual counting and differentiation of 100 white blood cells/smear).

Plasma clinical chemical parameters were determined with a Roche Hitachi 917 Chemistry Analyzer (Hitachi, Tokyo, Japan) using commercial diagnostic kits (Diagnosticum LTD, Budapest, Hungary).

### Production and administration of the radiopharmaceutical tracer (^18^F-fluorodeoxyglucose, ^18^F-FDG)

The radiopharmaceutical tracer (allowance number: https://ogyei.gov.hu/gyogyszeradatbazis&action=show_details&item=133670) was produced using a TRASIS AllInOne universal radiochemistry synthesizer with a TRASIS S4000-7558 FDG (Trasis, Liége, Belgium) cassette in the early morning hours of the day of the measurements. The planned dose amount was stored in a sterile, rubber-sealed boro-silicate ampule isolated inside a shielded lead canister.

Just before the injection (into the anaesthetised animal), the radiopharmaceutical tracer was drawn into a B Braun Omnifix 3 ml Luer Lock syringe by hand. The dose amount (ca. 100 MBq / 2 ml) was measured with a daily-calibrated CURIEMENTOR 4 isotope calibrator (PTW, Freiburg, Germany). The syringe was inserted inside MR-compatible, tungsten syringe shield (Biodex W/Glass 3 ml, Loc ID: MA139, 007-961, NY, USA). The shielded syringe was carried in-house in an additional lead canister for additional radioactive protection.

### Anaesthesia, radiotracer administration and PET MR hybrid image acquisition

The PET MR imaging was performed before and after the 14-day mycotoxin exposure, on the same rabbit experimental cohort/identical individuals. Animals underwent PET MR imaging under anaesthesia induced with 3% Isofluran (Abbott Lab., IL, USA) via a small animal narcotic mask. To avoid urine (and ^18^F-FDG) contamination of the scanners, animals wore commercial human baby diapers during the scanning protocol.

The right hind leg was cannulated via the external iliac vein with an intravenous catheter of a Vasofix Braunüle® 20Gx1 ¼” (B. Braun Melsungen AG, Melsungen Germany).

Examinations were performed using a hybrid PET MR scanner (Biograph mMR, Siemens Healthcare GmbH, Erlangen, Germany). Blood glucose levels were checked before tracer injection to ensure that rabbits were ca. euglycemic. The rabbits received an administration of 100 MBq activity/individual of ^18^F-FDG (single tracer injection) intravenously, using an MR compatible, tungsten syringe shield (Biodex W/Glass 3 ml, Loc ID: MA139, 007-961), with 20 ml saline flush. The radiotracer was administered in a 2 ml bolus, containing the ca. 100 MBq dose.

### ^18^F-FDG PET MR image acquisition and reconstruction

^18^F-FDG PET MR image acquisition was performed on a 3T hybrid PET MR scanner (Biograph mMR, Siemens Healthcare GmbH, Erlangen, Germany) with a PET MR compatible Siemens mMR (24 channel) body coil and a Siemens mMR Spine (6 channel) spine coil. The rabbits were positioned in a head-first-prone position. After the T2 localization images, a GRAPPA MRAC image (TE = 3.56 ms, TR = 2.46 ms, flip angle = 10°, voxel size = 2.6 × 2.6 × 3.1 mm) was acquired for the MR-based PET attenuation correction. The μMAP used for attenuation correction of the PET images was calculated on-site by the Syngo MR E11 software (Siemens Healthcare GmbH, Erlangen, Germany), using a four compartment (air, water, fat, lung adaptive) model. After this, a 60 min list mode PET acquisition was started. The injection of 100 MBq ^18^F-FDG was administered as a bolus injection after 5 s from the start of the PET-measurement into the external iliac vein. After the PET scan, a bolus injection of 0.4–0.5 ml Gadovist solution diluted with 1 ml of water was administered, followed by a 20 ml saline flush. For anatomical purposes, a T1-weighted starvibe fs transversal post-contrast 3D sequence was acquired (TE = 1.97 ms, TR = 3.79 ms, flip angle = 9°, voxel size = 0.8 × 0.8 × 0.8 mm).

PET images (voxel size = 1.669 × 1.669 × 2.03 mm) were reconstructed retrospectively using the Ordinary Poisson OSEM algorithm with general purpose settings (iteration = 3, subset = 21) with model-based scatter correction and delayed event subtraction for random correction. A Gaussian kernel (FWHM = 4.0 mm) was used for spatial filtering during the reconstruction.

We used static and dynamic ^18^F-FDG images scaled to Standard Uptake Value (SUV) for PET image analysis, where the SUV was calculated as SUV = tissue activity (kBq/ml)/injected dose (kBq)/body weight (kg). Based on the injection time, a 40–60 min time window was used to reconstruct static images from the list-mode PET data. In contrast, the following time resolutions were used for evaluating the dynamic images: 10 × 3, 18 × 15, 10 × 60 and 9 × 300 s.

### Kinetic PET image analysis

We conducted a kinetic analysis that served to test the applicability of the reversible two-tissue compartment model, investigated using the blood supply from both the hepatic artery and portal vein for accurate kinetic modelling of ^18^F-FDG uptake of the liver, as proposed by Wang et al.^29^. To complete this task, we established eight spherical Regions of Interest (ROIs) with a 3–8 mm radius for the coarctatio aortae, lung, liver aorta, portal vein, left liver lobe, right liver lobe, renal cortex, and gut. During the image analysis stage, we utilized the T1-weighted MR images from the PET MRI system to identify the correct anatomical regions in the combined PET and MRI images. Using the delineated ROIs, we generated the appropriate Time Activity Curves (TACs) from the dynamic ^18^F-FDG PET image series.

### Static PET image analysis

In tumour diagnosis, the standardized uptake value (SUV) is the most widely used measure for quantifying metabolic activity. It is calculated as the ratio of tissue radioactivity concentration to the injected dose, normalised by BW. The maximum SUV (SUVmax) is the highest voxel value of the SUV found in the tumour. The SUVmax measurement of a tumour's metabolic burden is limited because it is based on only one voxel. Our study aimed to analyse the whole liver's entire glucose metabolism. To achieve this, we used parameters that combine glucose uptake volume and uptake intensity instead of SUVmax.

In ^18^F-FDG PET image analysis, two other parameters are commonly used: metabolic tumour volume (MTV) and total lesion glycolysis (TLG, but in further cases, this abbreviation is used to designate total liver glycolysis). MTV measures the size of the tumour with high metabolism using a specific SUV threshold, while TLG is calculated by multiplying the SUVmean and MTV. Using this concept, we have developed two new parameters based on the liver's metabolic activity: the metabolic liver volume ROI (MLV ROI) and the total liver glycolysis (TLG). We used a region-growing technique with a pre-defined threshold to generate the MLV ROI, a specific region in the liver where the standardized uptake value (SUV) is higher than a predetermined level. We were unable to apply any suggested/literature-based SUVmean or SUVpeak-based threshold successfully^30^. Nonetheless, the limited number of images allowed for a visual examination of the mask-fitting to the liver, which was fused on the PET and MRI images of the PET MRI scan. By utilizing this image-fusion technique, we established the threshold at 0.9 of the mean SUV value of a manually set spherical ROI. To calculate the TLG, we multiplied the volume of the MLV ROI (measured in cubic cm) by the SUVmean value of the MLV ROI^31^. Thus TLG considers not only the FDG uptake but also the liver volume.

**Figure 1:**
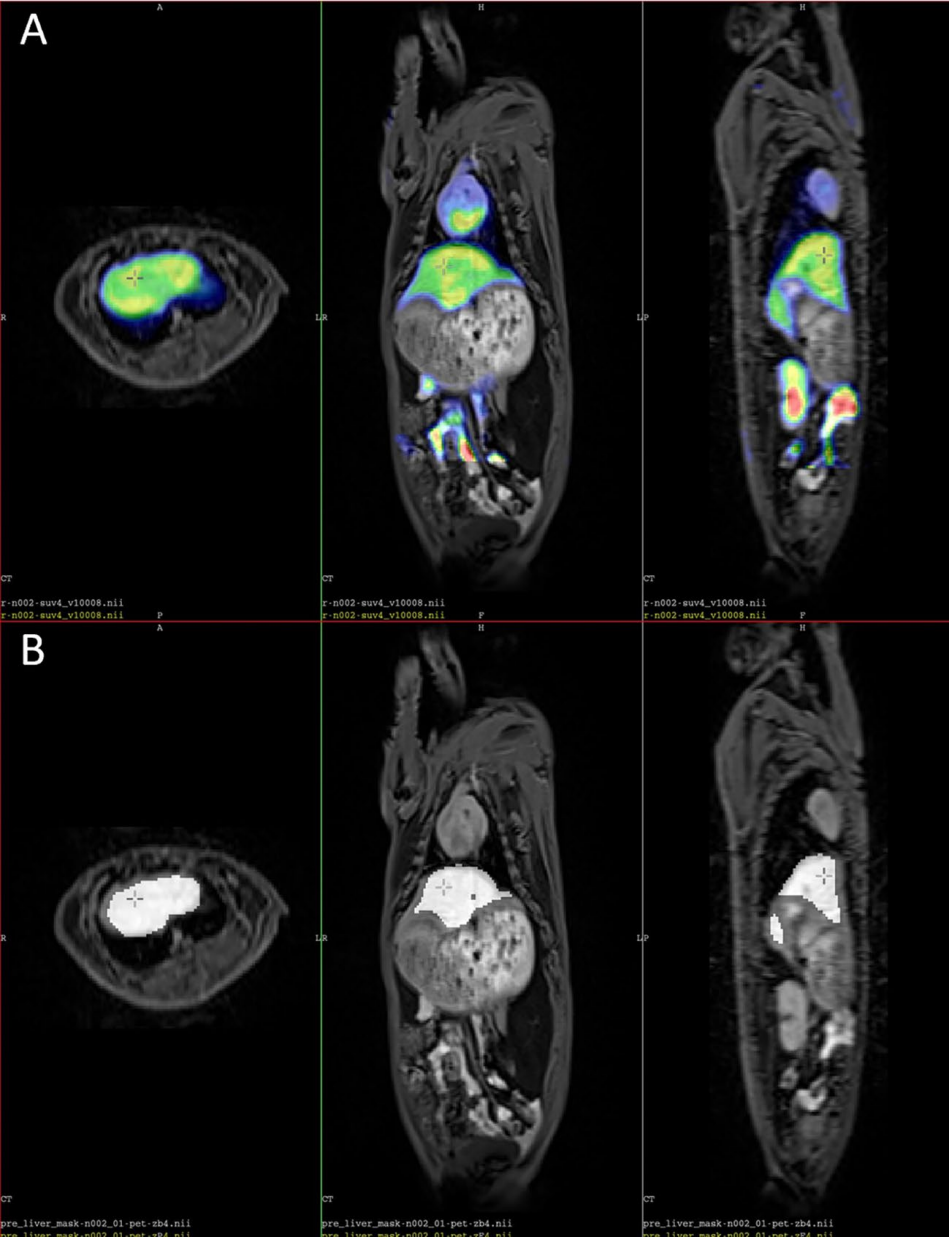
(**A**) Image fusion of ^18^F-FDG PET and T1 weighted MR images in three orthogonal sections. (**B**) The mean liver volume (MLV) generated with the region growing technique.

Figure 1 shows the fusions of ^18^F-FDG PET, T1-weighted MRI and the MLV ROI.

### Ethical allowance statement

The experiment was carried out according to the Hungarian Animal Protection Act regulations. The allowance number for the studies was SOI/31/00308-10/2017 (KA-2114) (date of approval: 27 March 2017), as issued by the National Food Safety and Animal Health Directorate, Hungary, Somogy County. Methods were carried out in accordance with relevant guidelines and regulations are reported in accordance with ARRIVE guidelines (https://arriveguidelines.org).

### Statistical evaluation

Bodyweight (BW), feed intake, clinical chemical and hematological data were evaluated with paired samples t-tests and Wilcoxon tests. For calculated indices and biochemical parameters relationship testing, Pearson correlation was calculated, using IBM SPSS Statistics for Windows (version 27.0)^32^.

## Results

### Growth and feed intake

**Figure 2:**
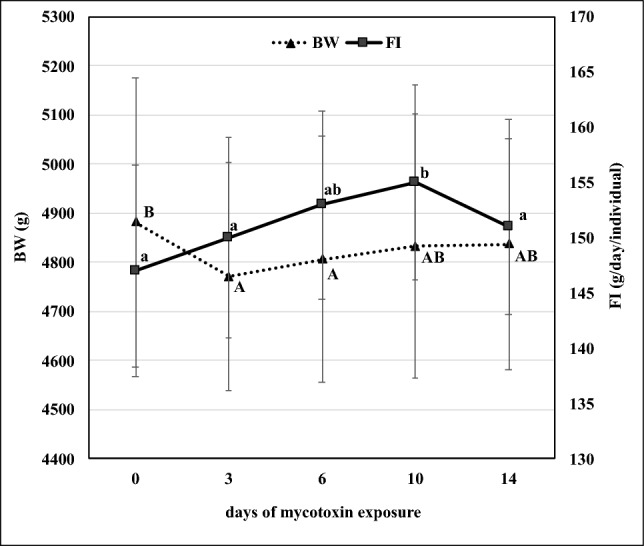
Changes of the mean bodyweight (BW, dotted line) and feed consumption (FI, continuous line) data during the mycotoxin feeding period (data points represent means ± SD, a, b: different letters indicate significantly different mean values between sampling events in FI. A, B: difference in BW between samplings (p ≤ 0.05).

After the first day (scanning), the BW decreased temporarily, being suggestive of a temporary growth retardation (Fig. 2). Feed consumption increased slightly, and after the 4th measurement (study day 10) it dropped to the baseline level.

### Blood plasma clinical chemistry

From the plasma *nitrogenous compounds*, total protein and within this, albumin concentration decreased significantly during the study period, while plasma creatinine concentration increased. Urea and uric acid concentrations were unchanged. Within the *lipids*, triglyceride (TG) and total cholesterol decreased significantly, meanwhile HDL cholesterol was unchanged. In *ions*, potassium (K) and total calcium (Ca) decreased during the study, while none of the other ions (Na, P, Cl, Mg) provided any significant change. Plasma *enzyme activities* showed a decrease in AST activity and an increase in gamma-GT and alkaline phosphatase (ALP). ALT, cholinesterase and creatine kinase (CK) levels remained unaltered (Table 2).

**Table 2:** The plasma clinical chemical parameters of the rabbits in the study (means ± SD).

Parameter	Control	FBs	P-value (Wilcoxon test)
	Mean ± SD	Mean ± SD	
total protein (g/l)	61.0** ± **2.35	57.0** ± **2.90	0.039
albumin (g/l)	36.2** ± **4.09	28.3** ± **2.07	0.043
urea (mmol/l)	5.96** ± **0.87	5.87** ± **0.82	n.s
uric acid (µmol/l)	10.0** ± **4.18	11.2** ± **3.43	n.s
creatinine (µmol/l)	82.8** ± **12.5	103.0** ± **12.3	0.043
triglyceride (mmol/l)	1.17** ± **0.33	0.54** ± **0.24	0.042
total chol. (mmol/l)	1.52** ± **0.36	0.60** ± **0.17	0.043
HDL chol. (mmol/l)	0.25** ± **0.10	0.38** ± **0.09	n.s
Na (mmol/l)	143.0** ± **1.87	146.0** ± **2.37	n.s
K (mmol/l)	7.81** ± **1.08	6.26** ± **0.79	0.043
Ca (mmol/l)	3.30** ± **0.12	3.08** ± **0.18	0.043
Ca corr. (mmol/l)	2.13** ± **0.12	2.07** ± **0.13	0.043
P (mmol/l)	1.10** ± **0.28	1.22** ± **0.21	n.s
Cl (mmol/l)	99.4** ± **2.30	99.5** ± **3.21	n.s
Mg (mmol/l)	1.12** ± **0.07	0.98** ± **0.08	n.s
Fe (µmol/l)	25.7** ± **9.92	19.0** ± **5.46	n.s
LDH (IU/l)	1600.4** ± **596.3	180.7** ± **123.2	0.043
AST (IU/l)	47.6** ± **16.8	16.3** ± **4.50	0.043
ALT (IU/l)	45.4** ± **10.4	47.7** ± **11.9	n.s
gamma-GT (IU/l)	3.00** ± **7.35	7.67** ± **1.75	0.043
cholinesterase (IU/l)	1032** ± **1454	969.2** ± **1222	n.s
lipase (IU/l)	470.0** ± **119.1	325.0** ± **62.7	n.s
ALP (IU/l)	7.00** ± **7.11	15.0** ± **4.20	0.042
CK (IU/l)	647.2** ± **213.4	446.3** ± **118.3	n.s

*ALP* alkaline phosphatase, *ALT* alanine transaminase, *AST* aspartate aminotransferase, *CK* creatine kinase, *gamma-GT* gamma-glutamyl transferase, *HDL* high density lipoprotein, *LDH* lactate dehydrogenase, *ns*: P > 0.05, not significant.

### Haemogram

Mean data on the cell distribution in the haemograms are given in Table 3. Irrespective of treatment (sampling), erythrocytes exhibited a mild anisocytosis, but no marked shape difference was identified. The leukocyte distribution, specifically that of heterophilic granulocytes and lymphocytes, varied between individuals, but fluctuations were slight. After FBs treatment, high and low proportional tendencies of heterophil granulocytes and lymphocytes were observed without statistical significance. The distributions of other leukocytes in the blood (eosinophil and basophil granulocytes, monocytes) were not significantly different before and after treatment.

**Table 3:** Qualitative leukocyte proportions (%) in rabbits exposed to 15 mg/kg FBs for 14 days.

Cell type	Control	FBs	P-value	Physiological range*
	Mean ± SD	Mean ± SD		
Heterophil granulocyte	32.4 ± 17.1	53.7 ± 12.3	n.s	20–75
Eosinophil granulocyte	1.20 ± 0.45	1.75 ± 0.96	n.s	0–5
Basophil granulocyte	0.40 ± 0.55	0.50 ± 0.58	n.s	0–10
Lymphocytes	63.8 ± 16.9	41.7 ± 12.5	n.s	30–85
Monocytes	2.00 ± 0.82	2.25 ± 1.26	n.s	0–10

*Washington and Van Hoosier^63^.

### PET MR hybrid imaging

**Figure 3:**
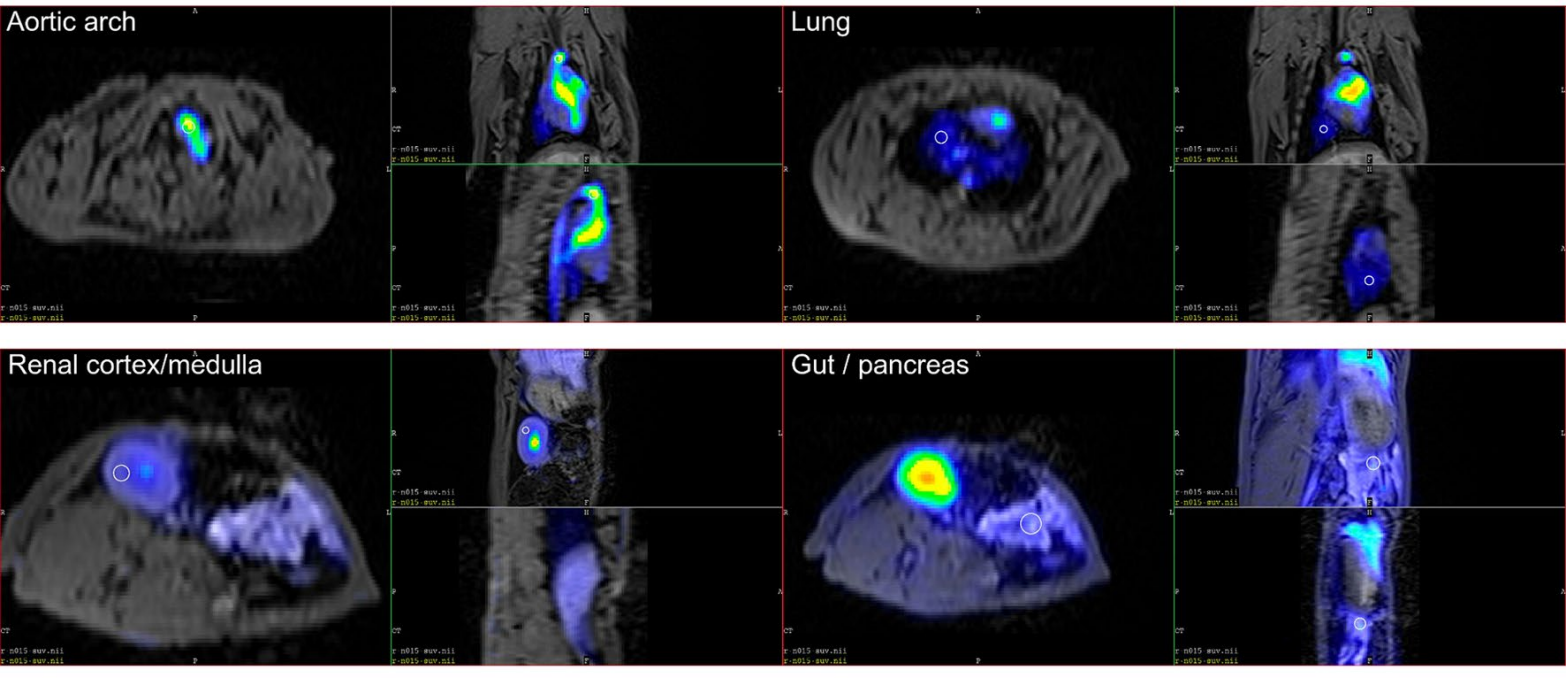
Fused ^18^F-FDG PET and T1-weighted MR images of a control-phase (before intoxication) rabbit that received 100 MBq ^18^F-FDG. The white circle contours show the section of 3D spherical ROIs.

Figure 3 shows the typical anatomical locations where ^18^F-FDG accumulation has been profound, i.e., in the digestive system, the aortic arch, and the kidney.

**Figure 4:**
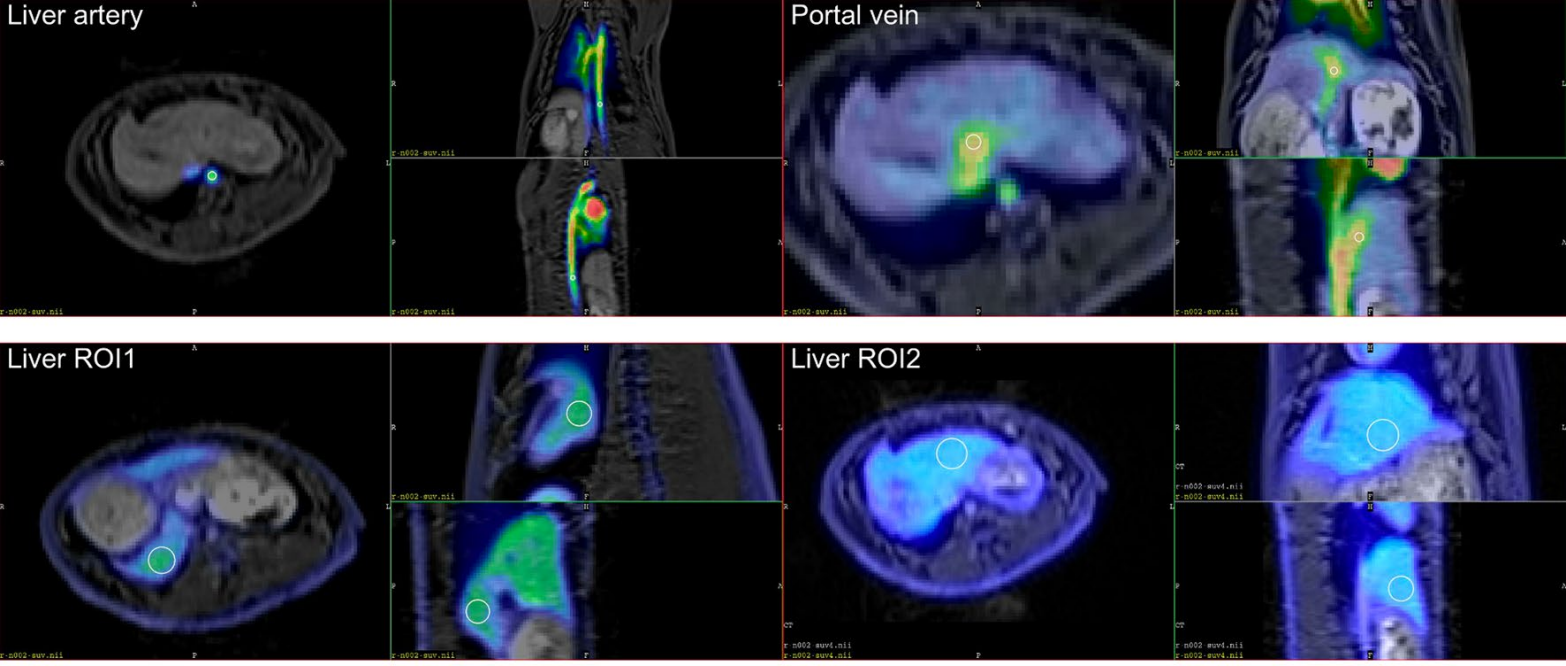
PET MR fused images (maximum intensity projection) of a control rabbit that received 100 MBq ^18^F-FDG. White circles are the hand-positioned ROIs.

For the same animal, the hepatic ROI system is given in Fig. 4.

### Time activity curves

**Figure 5:**
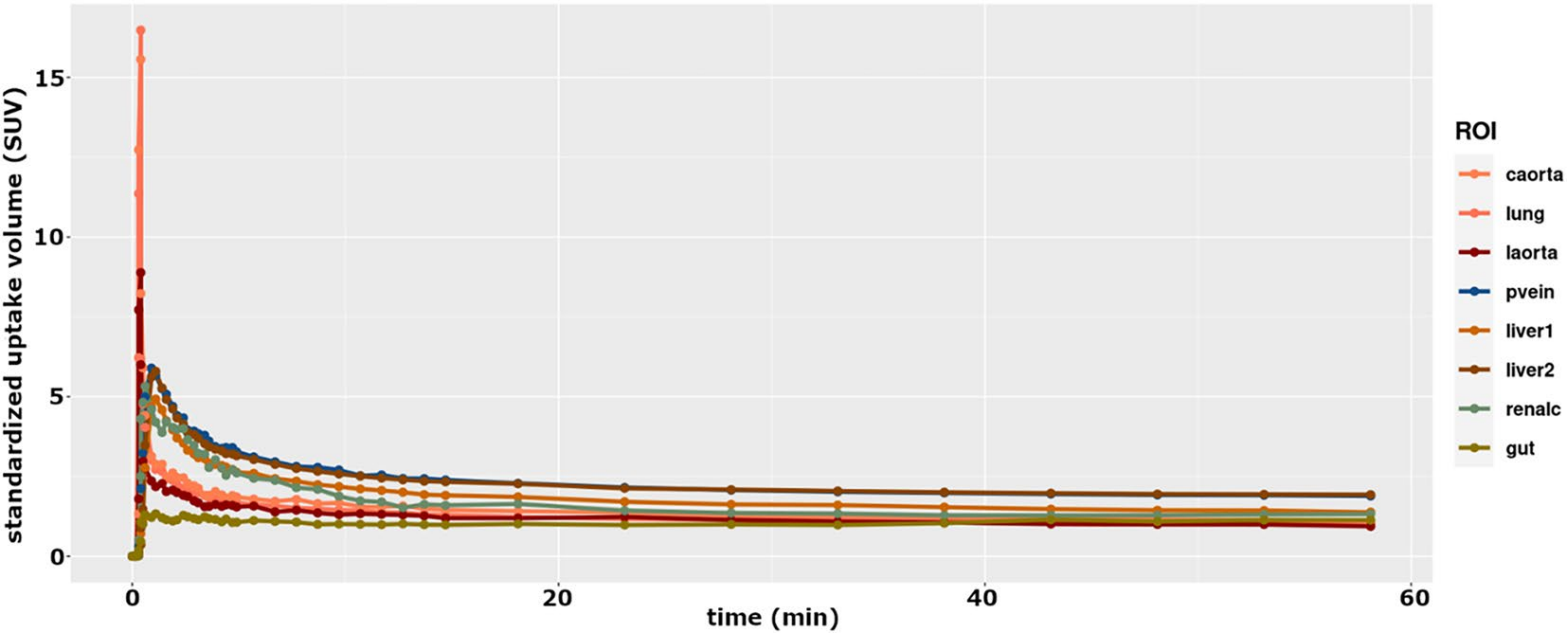
Time Activity Curves (TACs) generated by the spheric ROIs of a single animal. (caorta: coarctatio aortae, laorta: liver aorta, pvein: portal vein, renalc: renal cortex, liver1 and 2: left and right lobe, resp. SUV: standardized uptake volume).

Using the delineated spherical ROIs (see Fig. 4.), we generated Time Activity Curves (TACs) from all dynamic PET scans. Figure 5 shows one representative TAC system in the range of 0–60 min (Fig. 5).

This figure of TACs shows that ^18^F-FDG uptake in all areas of interest was rapid, reaching its maximum within 1 min, significantly faster than in humans (30–40 min), but after the maximum started a slight decrease of SUV values and the relative changes of the different ROIs' TAC minimized after 40 min. According to this observation, we applied the 40–60-min time window for generating static ^18^F-FDG images. During the ROI analysis, we found delineating artery and vein ROIs challenging; in some cases, we could not create proper carotid or liver arterial ROIs even using good anatomical information from MR images. This practice may be explained by the PET imaging technique's partial volume effect (PVE), i.e., the artery and vein size are lower below the 3.5 mm spatial resolution of the PET camera system.

### Metabolic liver volume and total liver glycolysis

**Figure 6:**
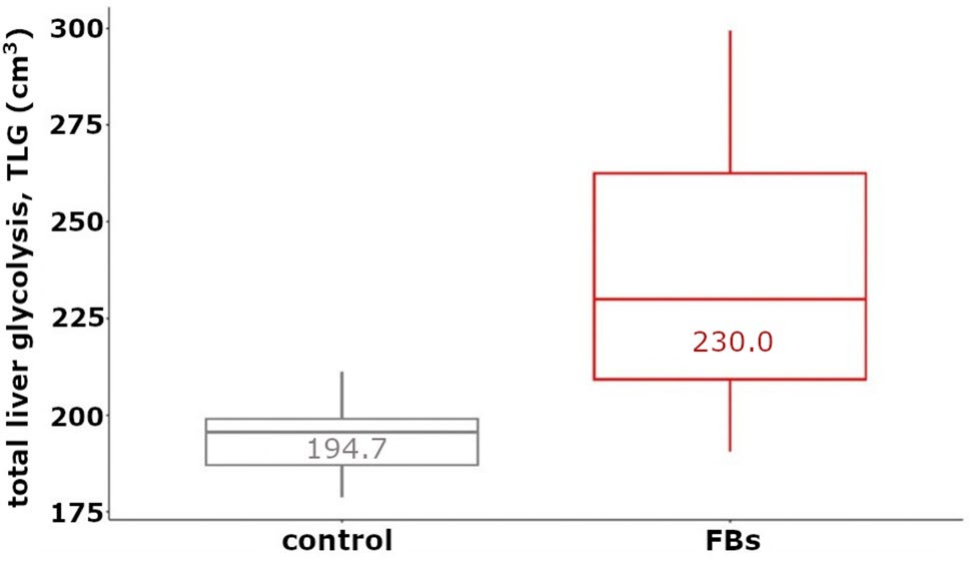
The increase of the TLG (total liver glycolysis) value of rabbits before and after the FBs treatment (Wilcoxon test: p < 0.04, data in the boxplot are median values).

Based on MLV and TLG calculations, the total hepatic ^18^F-FDG uptake and the difference (before and after intoxication) were calculated. Before and after intoxication group median values are shown in Fig. 6, providing a significant (p = 0.031) difference. The median MLV data were 100.4 cm^3^ and 116.2 cm^3^, respectively, and the median of SUVmean values were 2.03, both in the control and FBs cases.

### Inter-relationship between hepatic glucose uptake and clinical chemistry

As a final step, the possible inter-relationship between blood clinical chemical (direct biochemical traits) data and TLG was analyzed with Pearson correlation analysis (Table 4).

**Table 4:**
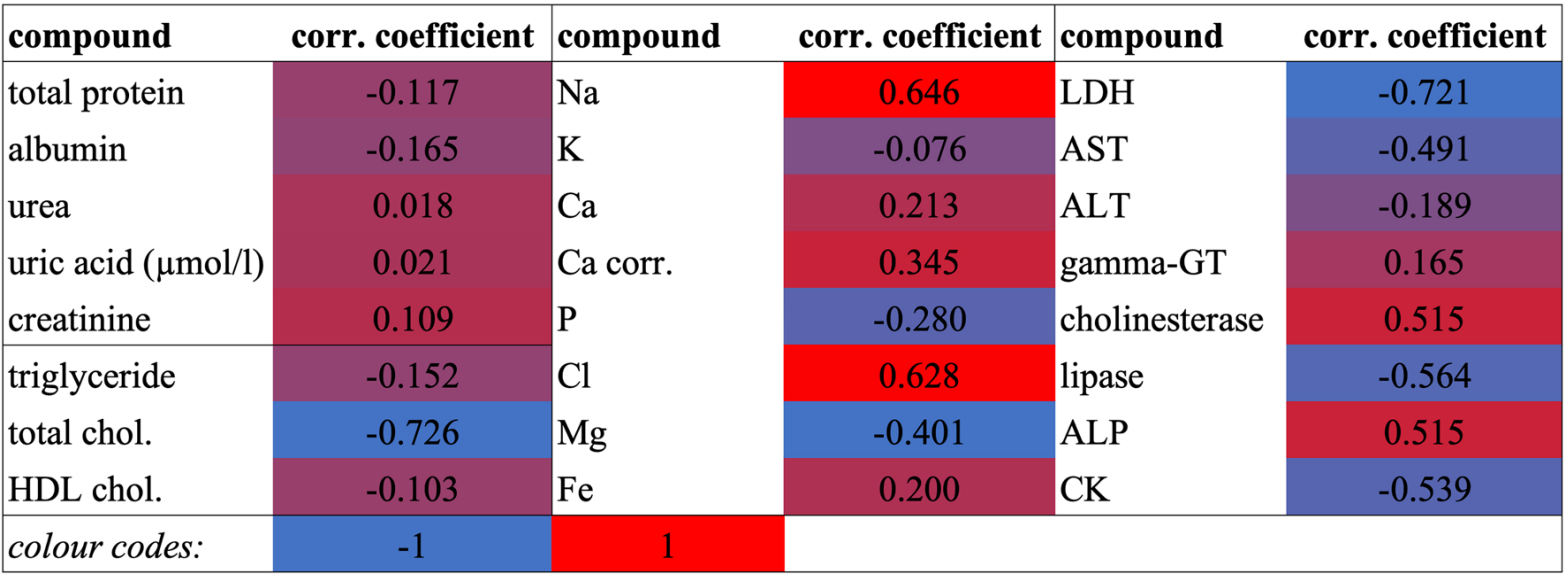
The Pearson correlation coefficients between TLG and the analysed clinical chemical endpoints.

*ALP* alkaline phosphatase, *ALT* alanine transaminase, *AST* aspartate aminotransferase, *CK* creatine kinase, *gamma-GT* glutamyl transferase, *HDL* high density lipoprotien, *LDH* lactate dehydrogenase.

The closest positive correlation of TLG (referring to the cases showing as well inter-group difference) was found with ALP, while the closest negative correlation was with total cholesterol, LDH and AST.

## Discussion

### Growth and feed intake

In a recent study, we fed a rabbit cohort (of the same breed, Pannon White) a feed contaminated with 10 and 20 mg/kg FBs, respectively^33^. The rabbits in the cited study were enrolled into a reproduction study (for 65 days), with minimal handling; these animals showed slow but balanced growth without fallback. In another FB_1_ intoxication study, 10 mg/kg FB_1_ for 4 weeks did not compromise rabbit buck growth^34^. Ewuola^35^ fed rabbit bucks FB_1_ (0.13, 5, 7.5, and 10 mg/kg diet) for 196 days and found no fallback in BW. At the same FB_1_ concentration range for 175 days, the onset of puberty was delayed by the two higher doses (i.e., 7.5 and 10 mg/kg), but the mycotoxin did not significantly influence bodyweight at puberty. Most probably, the BW loss recorded in the first 3 days shall be a treatment/handling (anaesthesia and subsequent PET MR imaging protocol) associated effect, and it was clearly visible that the growth curve continued after this initial fallback.

From the literature, the effects of FBs on FI seem controversial. Ewuola et al.^36^ (12.3 and 24.5 mg FB/kg diet for 5 weeks) reported a contradicting (decrease in FI) finding, a similar result has been reported by Szabó et al.^33^. Since our recorded high feed intake was temporary, it fell back to the baseline at day 14. This may be supported by the finding of Hafner et al.^10^, where 10 mg/kg dietary FB_1_ increased feed intake of growing rabbits temporarily (during the first week) compared to non-intoxicated controls.

### Clinical chemistry

#### Nitrogenous compounds

After 14 days of FBs exposure, total protein and albumin concentrations fell considerably in the plasma, whereas the plasma creatinine concentration increased, latter is a widely accepted indicator of fumonisin-induced nephropathy (in rabbits:^10^, in farm animals:^37^). Ewuola and Egbunike^36^ reported that in rabbits, serum total protein showed an FB_1_ dose-dependent concentration drop. The authors explained this with possibly altered dietary protein metabolism, including compromised digestibility and absorption of this nutrient. This has been more recently supported by Zeebone et al.^38^, where the apparent ileal digestibility of crude protein and more particularly that of arginine, histidine, and tyrosine was altered (exposure time × dose interaction) by 40 mg/kg dietary FBs for 7 and 21 days in piglets. In the present study, plasma total protein concentration values of both groups (control and FB_1_) fell into the physiological reference range (5.5–8.0 g/L), according to Mitruka and Rawnsley^39^. Indeed, at the dose applied, we did not prove either hypoproteinemia or hypoalbuminemia, and the unchanged levels of urea and uric acid in both groups refer to a balanced protein metabolism (supported by data of^36^), while creatinine referred to mycotoxin associated compromised renal function. As the ultimate reason of the findings on TP and albumin, we rather suppose altered/minimally compromised hepatic function (as indicated by AST and gamma-GT).

#### Plasma lipids

Within the lipid metabolites, TG and total cholesterol concentrations decreased significantly, as well as LDL cholesterol. These alterations refer to a very mild extent of FB intoxication. Since serum TG concentration is direct correlate of dietary fat intake, its discussion in relation to FBs is void. In contrast, relevant total cholesterol results of Ewuola^40^ provided a dose-dependent concentration increase in rabbits (0.1, 5, 7.5, and 10 mg/kg doses), and this hypercholesterolemic effect of FB_1_ is widely published in mammals^41^.

An earlier in vitro study by Gelderblom et al.^11^ referred to the strong compositional modification of hepatocellular membrane phospholipids, modifying the membrane structure and altering membrane cholesterol concentration. In multiple studies (e.g. Bondy et al. and Szabó et al.^42,43^) on FB_1_ effects on rats, blood plasma total cholesterol (of which free cholesterol is a major component) was reported to increase. However, fumonisin-associated hypercholesterolemia aetiology is not fully elucidated. According to Ridgway^44^, this effect is not a result of FB_1_-induced sphingosine- or sphinganine mediated inhibition of cholesterol esterification. Our results (total cholesterol decrease) hypothesize only a mild FB_1_ effect on the hepatic lipid transport mechanism (towards extrahepatic sites). Furthermore, our data do not support the presence of hypercholesterolemia since recorded levels remain within the physiological range (0.3–3 mmol/l), according to Harcourt-Brown^45^.

#### Enyzmes

From the plasma *enzymes*, AST activity decreased during the study, while gamma-GT and alkaline phosphatase (ALP) increased. Relevant literature refers to a profound hepatic malfunction in rabbits as an effect of FB_1_. According to Orsi et al.^46,47^, AST, ALT, gamma-GT and ALP are responsive. A possible reason for the lack of FB_1_-response of AST and ALT may be that those are not strictly liver-specific. In contrast, gamma-GT is a sensitive indicator of liver cell injury and already a single FB_1_ gavage dose (at 630 mg/kg feed dose) increased its activity in rabbits^46^. Hepatic injury was also demonstrated by a significant loss of body and liver weight, and authors interpreted ALP and gamma-GT levels as indicators suggestive of hepatic and/or biliary injury, sharing a common point with our results. Orsi et al.^46^ reported that a single FB_1_ dose vs. prolonged exposure (1.5 mg/kg for 21 days) induced a shift in organ-level intoxication, namely an early phase that is typically nephrotoxic, and this coupled with hepatotoxicity later, as corroborated by the findings of Gumprecht et al.^48^ and supporting the creatinine results.

*Analysing the metabolic and enzymatic data comprehensively*, we suppose mild hepatotoxic effect of dietary FBs (hepatocellular damage and consequent intracellular enzyme leakage: ALP and gamma-GT) with probably compromised hepatic albumin synthesis, and as well nephropathy development (increased creatinine level and ALP activity) with probable urinary protein loss (total protein and albumin level decrease in the serum).

### Haemogram

The effects of fumonisins on rabbit erythrocytes are not yet fully elucidated; the results of earlier studies are controversial. In some cases, FB_1_ induced^36,49,50^ and could not induce^36,46^ proportional haemogram shifts. Anisocytosis is a condition that frequently occurs in rabbits, resulting in a cell diameter of up to one-quarter of the normal erythrocyte. Szabó et al.^51^ demonstrated that a 10 mg FB_1_/kg diet for 28 days markedly elevated rabbit erythrocytes' total Na^+^/K^+ ^ATPase activity. Szabó et al.^52^ confirmed similar findings in piglets fed a 30 mg FB_1_/kg diet for 21 days. The authors proposed that alterations in sodium pump activity and membrane lipids occurred as a consequence of the inhibition of ceramide synthase activity, a characteristic FB mode of action. The findings of Szabó et al.^51,52^ postulate that FB1 may alter erythrocyte surface/volume ratio and osmotic properties. In this study, mild anisocytosis was observed, suggestive of the negligible effect of FBs on erythrocyte morphology. Our findings corroborate those of Ewuola et al.^36^, who found that a 24.56 mg FB/kg diet for 5 weeks did not alter the haematological parameters of rabbits^36^.

To our knowledge, the in vivo effects of FBs on blood *leukocyte* counts are rarely discussed, especially in rabbits. From the available literature on mice^53^, swine^54^, and humans^55,56^, the effects of FB on the immune system vary according to species, sex, mode of toxin application, exposure period and mycotoxin dose. Although our findings showed only tendencies in leukocyte distributions, no statistically significant difference was proven. This finding implies that FBs have no or only a negligible effect on leukocytes. However, further investigations are needed to determine the implications of FBs on leukocytes following a prolonged exposure approach.

### PET MR hybrid imaging

In the 1^st^ step of image analysis, we primarily focused on seeking the anatomical locations where radiotracer uptake is the most intense in the rabbits, aided by the PET MR hybrid imaging technique.

Relevant rabbit-based literature is scarce, but Probst et al.^57^ reported basically similar ^18^F-FDG biodistribution data in a rabbit model developing papillomavirus-induced tumors. More specifically, healthy skin, blood, liver, spleen, kidneys, heart, lung and fat were noted before infection and tumor development. In contrast, during tumor development, the tumor itself and the liver provided increased relative uptake of ^18^F-FDG. Similar to our results, skeletal muscle was found to provide low ^18^F-FDG uptake, and it was not changed during the study interval. Basically, similar results were described by Li et al.^27^, in that kidneys, spleen, liver and genitals were primary glucose uptake sites. Based on the portal vein and liver ROI data, we found that liver ^18^F-FDG is a determinant proportion of the total uptake and might sensitively reflect the mycotoxin-induced metabolic disturbance.

### Time activity curves

The *primary goal of kinetic analysis* was to check the ^18^F-FDG uptake of different tissue types and to determine the time needed to reach the uptake maximum. Indeed, this has been found to be very rapid (below 2 min). Our results are consonant with those of Probst et al.^57^ in rabbits, reporting peaking ^18^F-FDG uptake immediately after administration (below ~ 5 min), and explaining this result with the high blood-pool concentration of FDG. Probst et al.^57^ used 55-min acquisition time and described an early peak and a slight but stable decay during this interval for hepatic glucose uptake. We used 5 min only, but reported identical findings. Checking the data in Fig. 5, it is clearly visible that blood, arteries and veins showed the quickest and highest uptake, and from the organs, the liver was the primary uptake site (followed by the lung and intestines), being consonant with the findings of Probst et al.^57^, where liver uptake rate exceeded that of the tumour and skeletal muscle as well.

The *second aim of the kinetic analysis* was to check the usability of the two-compartment tissue model of the liver^29^. To use this model, two blood TACs (one from the liver artery and one from the portal vein) and two liver TACs (one from the lesion and one from the reference ROIs) were required. In all cases, we successfully delineated the two tissue ROIs and the portal vein. However, we encountered difficulty locating the liver artery TAC in almost all cases. This is due not only to the partial volume effect but also to the anatomical fact that the artery and the aorta abdominalis are located too close. As a result, we could not separate the kinetics of the liver artery from the a. abdominalis kinetics, as the latter transported the bolus-injected ^18^F-FDG from the rabbit's leg to the heart.

After analyzing the kinetic data in detail, we concluded that the liver FDG uptake cannot be accurately quantified using any tracer kinetic model. To successfully complete similar experiments it is crucial to have more standardized procedures for administering ^18^F-FDG injections and selecting the input location.

### Metabolic liver volume and total liver glycolysis

Standardized uptake value, metabolic tumor (liver) volume, and total lesion (here: liver) glycolysis are considered as efficient prognostic factors in many cancerous cases. Basically, we intended to seek possible correlations between a well-predictable hepatic malfunction and the volumetric, functional metabolic parameters. Since the hepatotoxic effect of FBs in rodents is already well predictable, but hepatic glucose uptake has not yet been addressed as a function of FB dosage, we tested our hypothesis that FBs may increase glucose uptake rate. A specific point of our study was that MLV and TLG are used in tumor staging, while we used not only intra-individual but also study-cohort level mean values successfully. The FBs induced slight hypermetabolic conditions of the rabbit liver, which could be well characterized with TLG.

### Inter-relationship between hepatic glucose uptake (TLG) and clinical chemistry

We handle this joint evaluation of direct clinical-chemical and indirect, molecular imaging data as the most important part of our work. Our toxicological practice in rodents (e.g. Szabó et al.^43^ in rats), more specifically in rabbits (e.g. Hafner et al.^10^) already clarified numerous clinical chemical consequences of fumonisin B intoxication. Indeed, the link between hepatic glucose uptake and fumonisin B intoxication is quite direct, namely sphingolipids, as regulatory molecules correlate with insulin resistance development^18^. According to Babenko and Kharchenko^18^, ceramides are the most effective insulin signal transduction inhibitors. The inhibition of hepatic ceramide accumulation plays an important role in normalizing glucose homeostasis^18^, and this inhibitory effect of FBs is well-known.

The TLG value increased significantly as a result of FBs intoxication, referring to a shift in glucose metabolism. We detected a fallback in FI, and thus, the improved glucose uptake ability/intensity of livers seems paradoxical in that the dietary nutrient intake was lower after the intoxication than before. A possible explanation for this may be the FBs induced alteration of the intestinal absorption of distinct nutrients. This has been shown in pigs by Lessard et al.^22^, specifically for jejunal glucose transport, where FB_1_ markedly elevated glucose absorption. The authors explained the augmented glucose transport with a possibly increased cellular density of glucose co-transporter sites in the enterocytes’ apical membrane. Lessard et al.^22^ reported FB_1_-induced absorptive and secretory physiological changes, primarily affecting sodium-dependent glucose absorption. We have no ready hypothesis for our finding as to why plasma Na (and Cl) ion concentrations provided an important and positive correlation with TLG (meanwhile, the concentrations were unchanged within study groups), but the role of Na in glucose absorption is well-established^58^. Moreover, the results are consonant with our findings in rats^43^, where 5 and 10 days FB_1_ dosage decreased plasma glucose levels, even besides absolute and relative liver weight reduction. Additionally, the results on Na-dependent transport intensification echo our earlier finding on rabbits, where red cell Na-pump intensity was significantly increased by dietary FB_1_ after 4 weeks^51^. Based on the present results, we are unable to make a clear distinction whether possible alterations of hepatic sphingolipid homeostasis (see Introduction) and/or altered (intestinal) glucose absorption (or transport) may stand behind the new observation on FBs-induced elevated hepatic glucose uptake.

In this study, in slight contrast with earlier FB feeding treatments, only mild intoxication was induced, and there were only a few FB-responsive biochemical endpoints providing characteristically sensitive intoxication response, primarily in terms of inter-group difference (ALP, gamma-GT). These two metabolites provided as well correlations with the livers’ molecular imaging-based functional parameter, TLG (Table 4).

*ALP* activity elevation in serum is correlated with the presence of disturbances of its bone, liver, or intestinal expression sites^59^. It is not possible to distinguish among different isoforms based on the plasma activity assay performed, but a very intriguing question arises as to why ALP is systematically elevated as a consequence of FBs dosage (pig:^60^, rodents, hepatic expression:^23^). Sharma et al.^59^ described a relationship between plasma ALP increase and hepatic malfunction, e.g. bile duct obstruction. In this in vivo study (without histopathology), the significantly increased gamma-GT activity is the most important supporting alteration, which is a typical response in FB_1_ intoxication (e.g. Ali et al.^60^).

Once referring to hepatic malfunction, *gamma-GT* is a marker of oxidative stress and cholestasis in rabbits^46^ and provided a TLG association. In earlier studies on rabbits, Gumprecht et al.^48^ reported mild hepatic necrosis, hepatocyte vacuolation, and bile stasis. Gamma-GT is a less specific FB_1_ toxicosis indicator but has been reported to increase in rats^61^, mice^62^, and rodent species with expressed renal sensitivity to fumonisins.

## Conclusions/highlights

The applied, relatively mild intoxication did not lead to a marked hepatotoxic effect (AST and gamma-GT were the most liver-specific responsive endpoints) in the rabbits, but hepatic function shift was found to be sensitively detected with molecular imaging. It is essential to note that our study is constrained by small population size (n = 4 bucks) and a short exposure duration of 14 days, which may limit the detection of subtle effects.

^18^F-FDG PET MRI has been found to be a sensitive technique for detecting slight (intoxication-associated) alteration in the total hepatic glucose uptake in rabbits, an optimally body-sized rodent model for toxicological studies (with minor anatomical limitations).

## Data Availability

Data will be made available upon request from the corresponding author, András Szabó, szabo.andras@uni-mate.hu.
